# Evolution of Clinical Features in Right Ventricular Outflow Tract Obstruction: Evidence From an Echocardiographic Database in China

**DOI:** 10.31083/RCM39701

**Published:** 2025-09-24

**Authors:** LiYun Su, DanQing Hu, TingXiang Lan, Xiang Zhang, Shuang Liang, QianYao Lai, JinGuo Li, XuDong Sun, Jun Fang

**Affiliations:** ^1^Department of Cardiology, Fujian Medical University Union Hospital; Fujian Cardiovascular Medicine Center; Fujian Institute of Coronary Artery Disease; Fujian Cardiovascular Research Center; Fujian Medical University Heart Center, 350001 Fuzhou, Fujian, China; ^2^School of Health, Fujian Medical University, 350122 Fuzhou, Fujian, China; ^3^Department of Ultrasound, Longyan First Hospital Affiliated to Fujian Medical University, 364000 Longyan, Fujian, China

**Keywords:** right ventricular outflow tract obstruction, pulmonary stenosis, pulmonary regurgitation, transcatheter pulmonary intervention

## Abstract

**Background:**

Long-term right ventricular outflow tract dysfunction can lead to right and left heart failure. Nonetheless, current data on the clinical characteristics of the right ventricular outflow tract obstruction (RVOTO) in China remain limited. This study analyzed the evolving trends in the observed proportion, etiology, spectrum, and interventions for RVOTO over the past 18 years from a single-center echocardiographic database.

**Methods:**

A total of 10,234 RVOTO cases (17,451 records from 2003 to 2020) were included in the transthoracic echocardiography database in China. The RVOTO cases were divided into eight groups according to the different obstruction sites and disease types. Subsequently, RVOTO was categorized into three types: congenital, iatrogenic, and neither congenital nor iatrogenic. Moreover, congenital RVOTO was further classified into simple and complex congenital RVOTO. Next, we calculated the proportion of RVOTO patients who had received interventions. We analyzed the proportions of different types of RVOTO and the variation tendency.

**Results:**

During 2003–2008, 2009–2014, and 2015–2020, the observed proportion of RVOTO cases in the total echocardiographic cases decreased (3.2%, 2.1% and 1.8%, respectively; *p *< 0.001); the proportion of RVOTO with a congenital etiology also reduced, though as the dominant cause; meanwhile, the proportion of simple congenital RVOTO increased (48.5%, 52.4% and 67.3%, respectively; *p *< 0.001). As the two most common types of RVOTO, the proportion and number of valvular pulmonary stenosis (PS) increased, while the proportion of Fallot cases decreased. The number of RVOTO cases receiving surgical or transcatheter intervention and experiencing intervention-related severe pulmonary regurgitation (PR) or residual peripheral PS increased, although with a low probability of reoperation.

**Conclusions:**

The clinical characteristics of RVOTO have undergone significant changes in China over the past two decades. After the RVOTO intervention, the increasing number of cases with severe PR or residual peripheral PS and a low possibility of reoperation signifies a future necessity for transcatheter pulmonary intervention.

## 1. Introduction

The right ventricular outflow tract obstruction (RVOTO) is mostly a congenital and relatively rare condition that has received little attention in the available literature. The disorder can occur at the sub-infundibular, infundibular, valvular, or supravalvular levels, with valvular pulmonary stenosis (PS) and tetralogy of Fallot (TOF) as the two most common types of RVOTO [1]. Due to RVOTO, a long-term right ventricular outflow tract dysfunction (RVOTD) can lead to the right and left heart failure, atrial or ventricular arrhythmia, and even sudden cardiac death, so RVOTO is often associated with significant morbidity and mortality [2, 3, 4]. Therefore, timely and optimal treatments are needed to avoid the pathological right ventricular remodeling in patients with RVOTO. Currently, surgery remains the main treatment for non-valvular PS, such as TOF, which usually requires the reconstruction of the right ventricular outflow tract (RVOT) [5]. However, the surgical efficacy is often compromised by artificial conduit degradation, pulmonary regurgitation (PR), residual peripheral PS, and repeated thoracotomy [6, 7]. Other than traditional surgery, the recent development of transcatheter interventional technology has provided other alternatives for RVOTO patients. Among them, percutaneous balloon pulmonary valvuloplasty (PBPV) has produced favorable short-, medium- and long-term effects on valvular PS, though PR remains a challenge and may require valve replacement [8, 9, 10]; percutaneous pulmonary valve implantation (PPVI), the first clinical percutaneous valve replacement technique [11] that has been proven feasible and safe [12, 13, 14, 15, 16], has been recommended as the preferred treatment to reduce the number of repeat surgical operations in patients with severe PR and RVOTD due to RVOT reconstruction or PBPV [1]; and balloon-expandable endovascular stents have also been shown to provide the efficacious treatment of residual peripheral PS after RVOTO intervention [17, 18, 19], and are also recommended by the guideline [1]. Meanwhile, despite the increased birth prevalence of total congenital heart disease (CHD) and simple CHD in China and in the U.S. owing to improved detection and diagnosis, the cases with complex CHD continue to decrease probably due to the impact of pregnancy termination in the past decades [20, 21, 22]. Though mostly congenital and relatively rare in CHD [22], RVOTO has recently attracted growing attention, thanks to the advance and availability of new technologies such as PPVI. However, data on the clinical characteristics of RVOTO remain limited in China. Existing data, primarily extracted from fragmented CHD studies, inadequately capture the full disease spectrum of RVOTO. To reveal the evolution of clinical features of RVOTO over time and identify the candidates for PPVI and pulmonary artery stent implantation, the present study attempted to analyze the changes in observed proportion, etiology, spectrum, and intervention of RVOTO over the past 18 years, based on an echocardiographic database in China.

## 2. Methods

### 2.1 Patient Population

We reviewed the transthoracic echocardiography (TTE) database from January 2003 to December 2020 at Fujian Medical University Union Hospital (one of the largest Cardiovascular Medical Centers in the southeast China). The clinical characteristics of all TTE-diagnosed RVOTO inpatients and outpatients were retrospectively analyzed. The definition of RVOTO is referred to American Society of Echocardiography’s, European Society of Cardiology’s, and other Guidelines [1, 23, 24, 25, 26]. The study was approved by the Ethics Committee of Fujian Medical University Union Hospital (Ethical Approval No.: 2020KJT078) and informed consent was exempted by the Ethics Committee due to its retrospective nature.

### 2.2 Classification of RVOTO

According to the different obstruction sites and disease types by echocardiography, RVOTO cases were divided into eight groups: (1) Subvalvular stenosis, including the simple sub-infundibular and infundibular stenosis; (2) Valvular PS; (3) Supravalvular PS, including stenosis in the main pulmonary trunk, the left and right pulmonary arterial (PA) branches (i.e., peripheral PS), and bifurcation; (4) Multiple site stenosis, involving two or more of subvalvular, valvular pulmonary, or supravalvular stenosis; (5) Fallot group, including trilogy of Fallot, TOF, and pentalogy of Fallot. Trilogy of Fallot includes infundibular, valvular, supravalvular RVOTO and/or branch PA stenosis, consequent right ventricular hypertrophy (RVH), and patent foramen ovale (PFO) or atrial septal defect (ASD). TOF is characterized by the following four features: a nonrestrictive ventricular septal defect (VSD); overriding aorta; infundibular, valvular, supravalvular RVOTO and/or branch PA stenosis; consequent RVH. Pentalogy of Fallot is TOF with PFO or ASD; (6) Pulmonary atresia; (7) Transposition of the great arteries (TGA), including complete and correct TGA with RVOTO; (8) Others, including hypertrophic cardiomyopathy, single ventricle, double outlet of right ventricular, tricuspid atresia, and other complex CHD. Groups (1)–(4) may coexist with other simple CHD, including patent ductus arteriosus (PDA), PFO, ASD, and VSD. All patients with PA and other cardiac malformations were included in Group (6).

Subsequently, the etiologies of RVOTO were divided into three categories: (1) Congenital, due to the presence of a disease or physical abnormality since birth; (2) Iatrogenic, caused by medical intervention or procedure; (3) Non-congenital/Non-iatrogenic, diseases or physical abnormalities caused by unclassifiable factors other than congenital or iatrogenic origins. Congenital RVOTO was further divided into simple congenital RVOTO (including subvalvular stenosis, valvular PS, supravalvular PS, and multiple site stenosis) and complex congenital RVOTO, including Fallot group, and so on [1].

### 2.3 Data Collection

Data on age, gender, the observed proportion, etiology, spectrum, and intervention (transcatheter interventional therapy or traditional surgical thoracotomy) in patients with RVOTO, were collected and analyzed in three time periods (2003–2008, 2009–2014 and 2015–2020). In patients with RVOTO receiving intervention, data on PR and residual supravalvular/peripheral PS were further collected and analyzed. The severity of PR is defined according to the American Society of Echocardiography’s native valves regurgitation guidelines and the prosthetic valves guidelines, including pulmonic valve, right ventricle size, jet size, ratio of PR jet width, deceleration time of the PR spectral Doppler signal, pressure half-time of PR jet, PR index and other indicators [26, 27, 28]. For patients who underwent multiple echocardiography examinations in each period, only the records of the most severe lesion or intervention were included for analysis. The data from the same patient may also appear in different time periods and were analyzed separately in this study. Due to the differences in the equipment and diagnostic criteria across the research periods, all data were re-evaluated by professionals.

### 2.4 Statistics Analysis

Categorical variables were expressed as frequency with percentage and continuous variables as median with interquartile range (IQR). The number of RVOTO was defined as absolute numbers of RVOTO cases (patients, not records) in each time period. The observed proportion of RVOTO was calculated corresponding to the whole echocardiographic population in each time period and expressed as RVOTO (n)/ population (n). The observed proportion of various RVOTO types was calculated corresponding to the whole RVOTO population in each time period and expressed as a certain RVOTO type (n)/ RVOTO (n). Statistical analyses were performed using the SPSS 26.0 software (IBM Corporation, Armonk, NY, USA) package. The comparison of multiple groups of continuous variables was analyzed by Kruskal-Wallis rank-sum test. The time trend of categorical data was examined by Linear-by-Linear Association. A *p *value of <0.05 was considered statistically significant.

## 3. Results

### 3.1 Observed Proportion of RVOTO Decreases Over Time

**Fig. 1.**
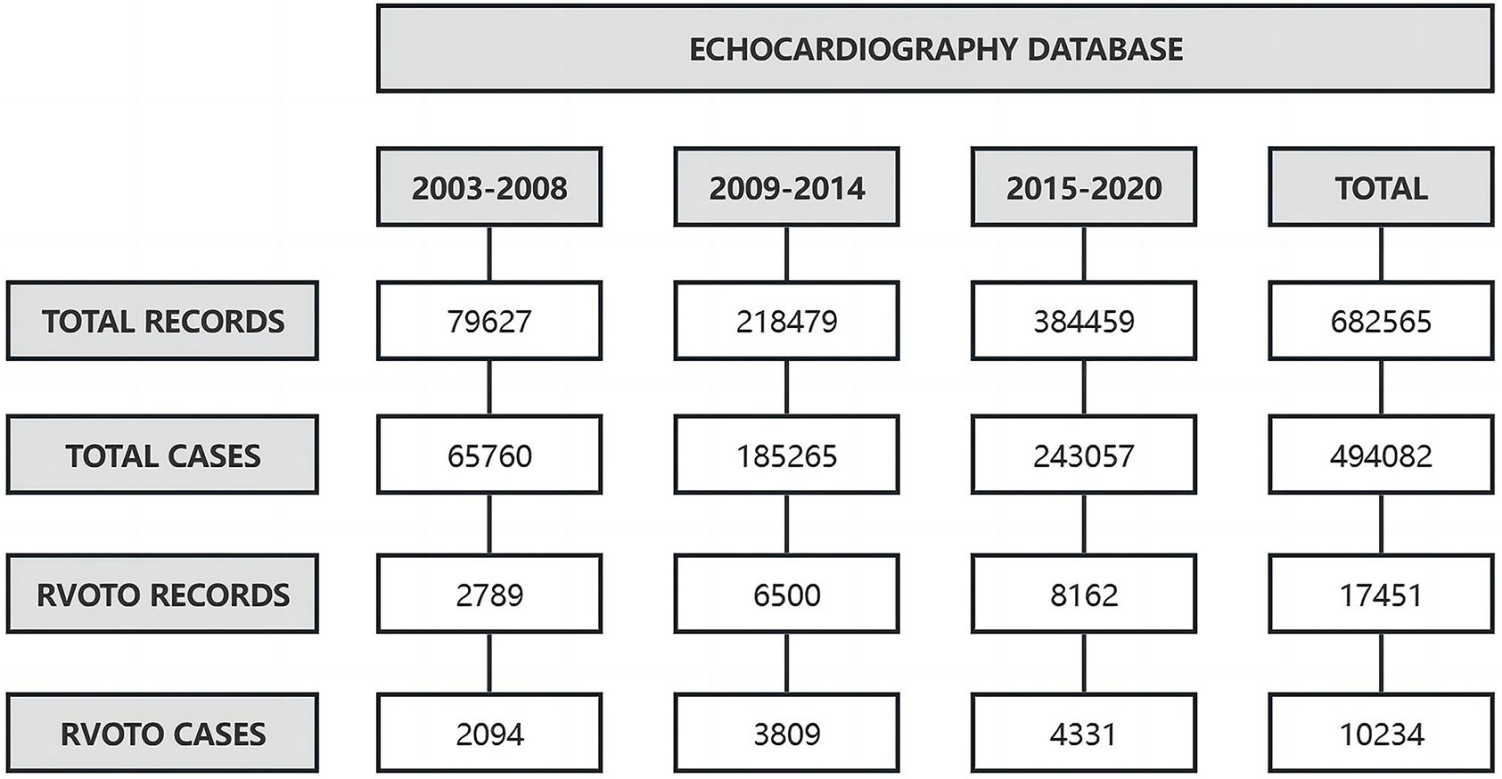
**The data extraction and change trend in the observed proportion of RVOTO over time**. Across the periods of 2003–2008, 2009–2014, and 2015–2020, the observed proportion of RVOTO in total cases significantly decreased, although the number of RVOTO cases increased. RVOTO, right ventricular outflow tract obstruction.

A total of 494,082 cases/patients (682,565 records from January 2003 to December 2020) were found in the TTE database of Fujian Medical University Union Hospital, of which 10,234 RVOTO cases (17,451 records) were included in the analysis. For the periods of 2003–2008, 2009–2014, to 2015–2020, the observed proportion of RVOTO showed a linear decreasing trend (3.2%, 2.1% and 1.8%, respectively, χ^2^ = 414.252, *p* < 0.001), despite the increase in the absolute case number (2094, 3809, 4331 cases, respectively) (Fig. 1).

During the three periods, there was a higher proportion of male RVOTO patients than that of females (57.3%, 53.7% and 50.8%, respectively, χ^2^ = 24.105, *p* < 0.001), with the proportion gap decreasing over time and the median age of RVOTO cases was 3.0 (0.5, 15.0), 2.0 (0.5, 12.0) and 2.0 (0.5, 13.0) years old (*H *= 14.019, *p *= 0.001), respectively. A comprehensive analysis of the three time periods showed that children (aged 0–3 years) accounted for more than half of the RVOTO patients and those aged ≤18 years accounted for about four fifths of the total. Meanwhile, the proportion and absolute numbers of RVOTO patients over 40 years old increased with time (5.4%, 7.7% and 11.0%, respectively, χ^2^ = 61.964, *p* < 0.001; 113, 293 and 476 cases, respectively) (Table 1).

**Table 1.** **Baseline characteristics of the patients with right ventricular outflow tract obstruction**.

		2003–2008	2009–2014	2015–2020	Statistic	*p* value
		N = 2094	N = 3809	N = 4331		
Sex, n (%)					χ^2^ = 24.105	<0.001
	Male	1199 (57.3)	2044 (53.7)	2200 (50.8)		
	Female	895 (42.7)	1765 (46.3)	2131 (49.2)		
Median age (years) (IQR)		3.0 (0.5, 15.0)	2.0 (0.5, 12.0)	2.0 (0.5, 13.0)	*H *= 14.019	=0.001
Age, n (%)						
	≤1	800 (38.2)	1654 (43.4)	1867 (43.1)		
	1–2	195 (9.3)	350 (9.2)	319 (7.4)		
	2–3	95 (4.5)	210 (5.5)	241 (5.6)		
	3–7	242 (11.6)	453 (11.9)	549 (12.7)		
	7–18	343 (16.4)	391 (10.3)	444 (10.3)		
	18–40	306 (14.6)	458 (12.0)	435 (10.0)		
	40–65	91 (4.3)	239 (6.3)	374 (8.6)		
	>65	22 (1.1)	54 (1.4)	102 (2.4)		

IQR, interquartile range.

### 3.2 Disease Spectrum of RVOTO Changes Over Time

**Fig. 2.**
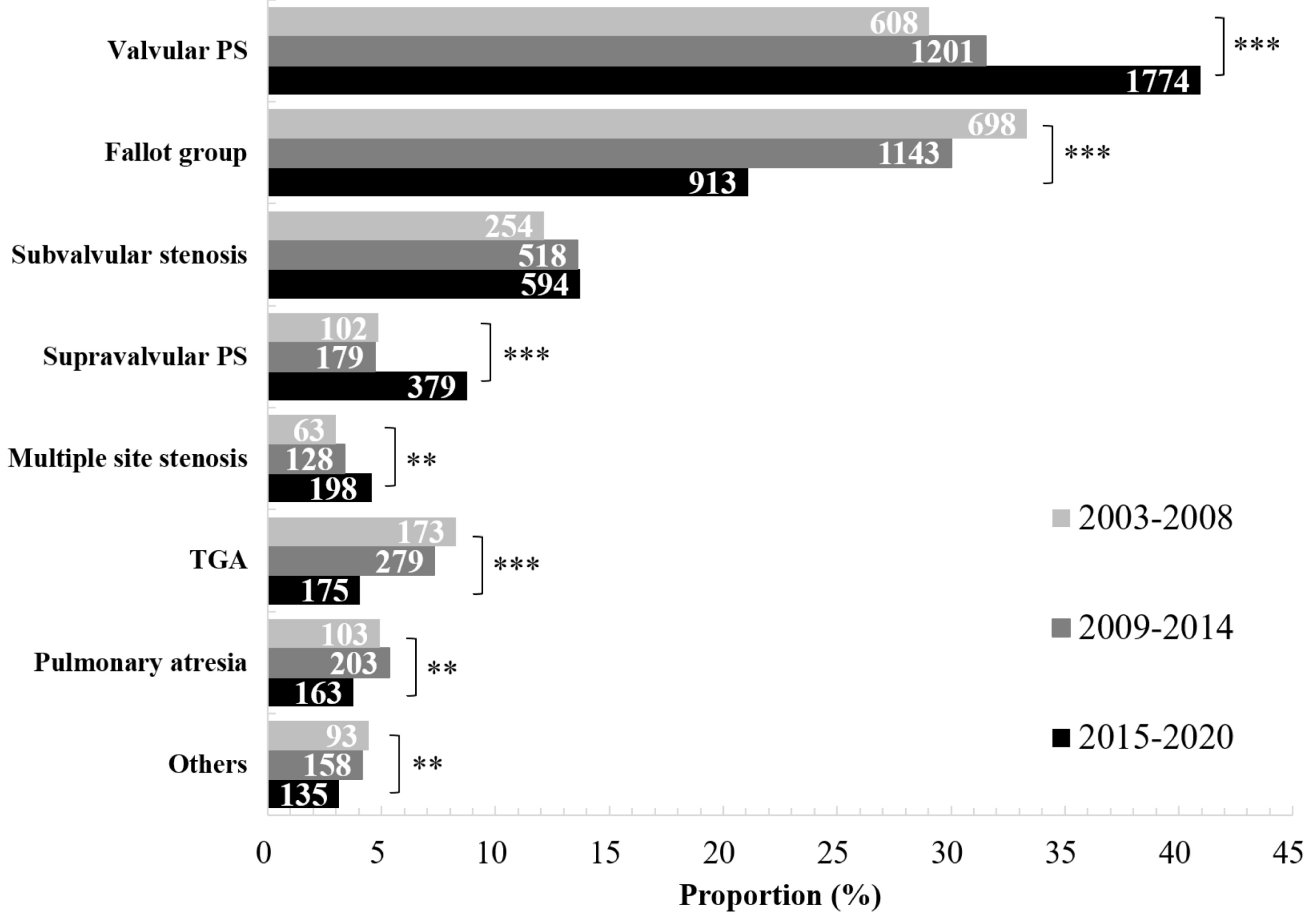
**The disease distribution and change trend of RVOTO over time**. The most common types of RVOTO were always valvular PS and Fallot group. During 2003–2008, the proportion of Fallot group ranked first; however, during 2009–2014 and 2015–2020, the proportion of valvular PS ranked first. The proportion in the figure refers to the proportion of each disease type in the total number of RVOTO cases in the same time period, and the length of the cross bar is in proportion to the percentage. The values in the cross bars represent the absolute number of observations of each type (Note: the length of the cross bar is not proportional to these values.). PS, pulmonary stenosis; TGA, transposition of the great arteries; RVOTO, right ventricular outflow tract obstruction. **p < 0.01, ***p < 0.001.

In each time period (2003–2008, 2009–2014, and 2015–2020), the most common types of RVOTO were always valvular PS, Fallot group, and subvalvular stenosis. Over time, the proportion of valvular PS, supravalvular PS, and multiple site stenosis increased (valvular PS: χ^2^ = 108.489, *p <* 0.001; supravalvular PS: χ^2^ = 49.190, *p* < 0.001; multiple site stenosis: χ^2^ = 11.503, *p *= 0.001), while Fallot group, pulmonary atresia, TGA and others decreased (Fallot group: χ^2^ = 128.254, *p* < 0.001; pulmonary atresia: χ^2^ = 6.963, *p *= 0.008; TGA: χ^2^ = 53.420, *p* < 0.001; others: χ^2^ = 8.333, *p *= 0.004). In detail, during 2003–2008, the proportion of Fallot group (33.3%) ranked first, followed by valvular PS (29.0%) and subvalvular stenosis (12.1%). However, during 2009–2014 and 2015–2020, the proportion of valvular PS ranked first (31.5% and 41.0%, respectively), followed by Fallot group (30.0% and 21.1%, respectively) and subvalvular stenosis (13.6% and 13.7%, respectively) (Fig. 2). Other types accounted for less than 10% over the three periods.

### 3.3 Trend of Etiology Distribution of RVOTO Changes Over Time

**Fig. 3.**
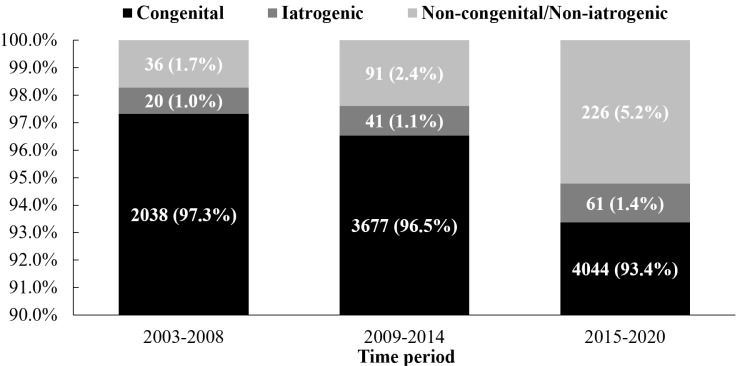
**The etiology distribution and change trend of RVOTO over time**. Congenital etiology was always the dominant cause of RVOTO. Over time, the proportion of congenital etiology decreased, and that of the iatrogenic etiology increased without statistical significance. The values before the parentheses represent the absolute number of observations of this type, and the values in parentheses represent the proportion of cases of this type in the total number of RVOTO cases in each period. RVOTO, right ventricular outflow tract obstruction.

In each time period, congenital etiology was always the dominant cause of RVOTO. However, over time, the proportion of congenital etiology decreased (97.3%, 96.5% and 93.4%, respectively, χ^2^ = 61.551, *p* < 0.001), while that of iatrogenic etiology and of non-congenital and non-iatrogenic etiology showed an increasing trend (iatrogenic: 1.0%, 1.1% and 1.4%, respectively, χ^2^ = 2.938, *p *= 0.087; non-congenital and non-iatrogenic: 1.7%, 2.4% and 5.2%, respectively, χ^2^ = 64.402, *p* < 0.001). The latter mainly included hypertrophic cardiomyopathy (26, 26 and 50 cases, respectively), tumor compression (2, 3 and 26 cases, respectively), and thrombosis or vegetations in the main pulmonary artery (3, 7 and 16 cases, respectively) (Fig. 3). In congenital RVOTO, over time, the proportion of simple congenital RVOTO increased (48.5%, 52.4% and 67.3%, respectively, χ^2^ = 237.888, *p* < 0.001), while that of the complex congenital RVOTO decreased (51.5%, 47.6% and 32.7%, respectively, χ^2^ = 237.888, *p* < 0.001). During the three periods, the number of iatrogenic RVOTO cases were 20, 41 and 61, respectively, and VSD patch or occluder protruding into RVOT consistently ranked first among iatrogenic causes (70.0%, 87.8%, 60.7%, respectively), followed by postoperative anastomotic stenosis. In the classification of stenosis sites, the most common atrogenic RVOTO is subvalvular stenosis (Table 2).

**Table 2.** **Classification of atrogenic RVOTO**.

Categary			Cases (n, % of total atrogenic RVOTO in the same time period)
2003–2008 (N = 20)			
	Etiology		
		VSD patch protruding RVOTO	14 (70.0)
		Postoperative anastomotic stenosis	3 (15.0)
		VSD patch vegetation	2 (10.0)
		Artificial duct compression RVOTO after anomalous origin of coronary artery surgery	1 (5.0)
	Type		
		Subvalvular	15 (75.0)
		Valvular PS	1 (5.0)
		Supravalvular PS	3 (15.0)
		Multiple site stenosis	1 (5.0)
	Severity		
		Mild	14 (70.0)
		Moderate	5 (25.0)
		Severe	1 (5.0)
2009–2014 (N = 41)			
	Etiology		
		VSD patch protruding RVOTO	28 (68.3)
		VSD occluder protruding RVOTO	8 (19.5)
		Postoperative anastomotic stenosis	5 (12.2)
	Type		
		Subvalvular	36 (87.8)
		Valvular PS	0 (0.0)
		Supravalvular PS	4 (9.8)
		Multiple site stenosis	1 (2.4)
	Severity		
		Mild	24 (58.5)
		Moderate	10 (24.4)
		Severe	7 (17.1)
2015–2020 (N = 61)			
	Etiology		
		VSD patch protruding RVOTO	32 (52.5)
		Postoperative anastomotic stenosis	20 (32.8)
		VSD occluder protruding RVOTO	5 (8.2)
		Aortopulmonary septal defect patch protruding RVOTO	2 (3.3)
		PDA occluder protruding RVOTO	1 (1.6)
		RVOTO plugged with ASD occluder	1 (1.6)
	Type		
		Subvalvular	38 (62.3)
		Valvular PS	0 (0.0)
		Supravalvular PS	21 (34.4)
		Multiple site stenosis	2 (3.3)
	Severity		
		Mild	37 (60.7)
		Moderate	15 (24.6)
		Severe	9 (14.8)

VSD, ventricular septal defect; PDA, patent ductus arteriosus; ASD, atrial septal defect; RVOTO, right ventricular outflow tract obstruction; PS, pulmonary stenosis.

### 3.4 Number of PR and Residual PS Cases After Intervention Increases Over Time

**Fig. 4.**
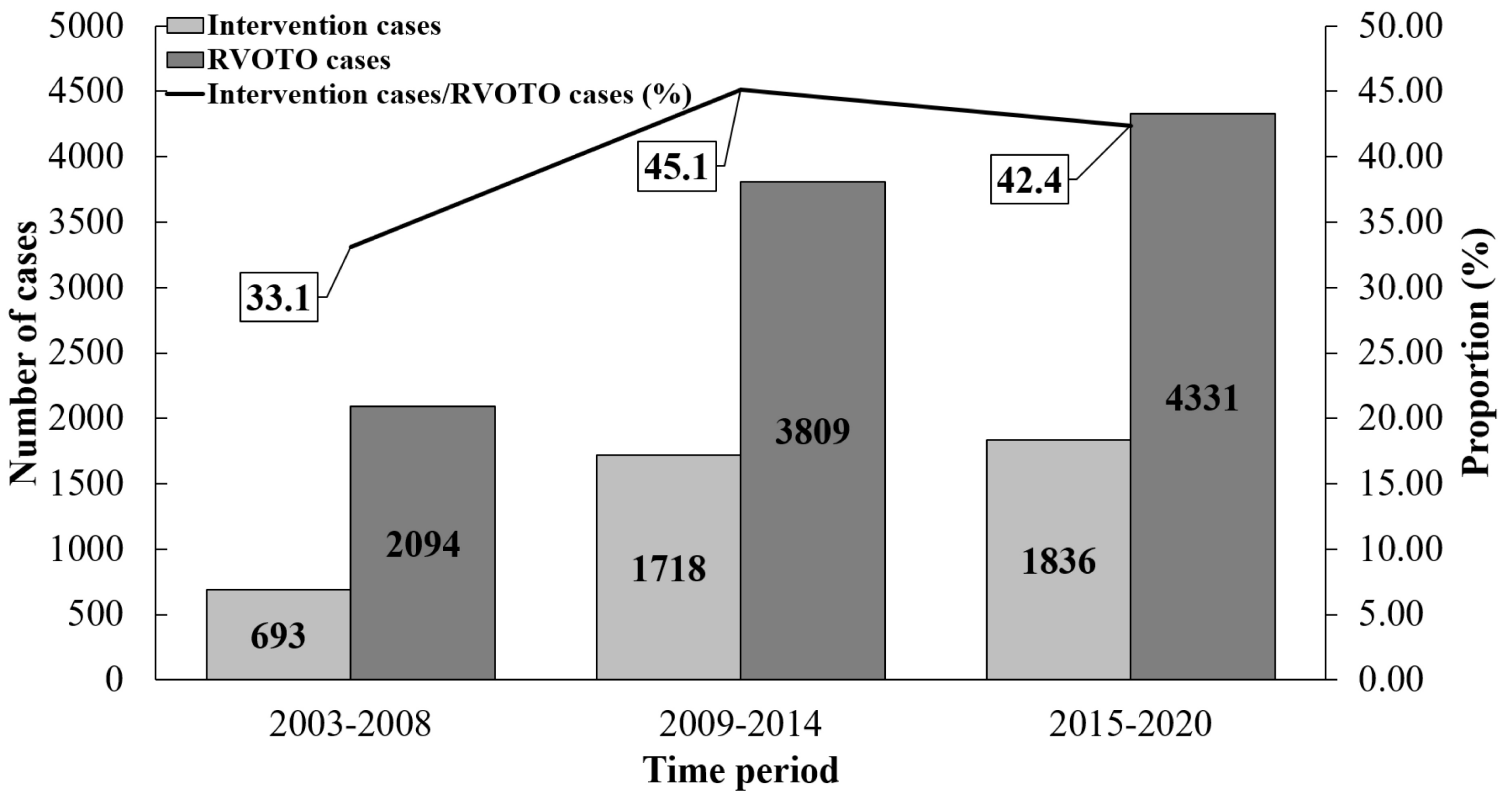
**The change trend of intervention proportion in RVOTO patients over time**. Over time, the absolute number of RVOTO cases receiving intervention (surgical or transcatheter) increased. RVOTO, right ventricular outflow tract obstruction.

**Fig. 5.**
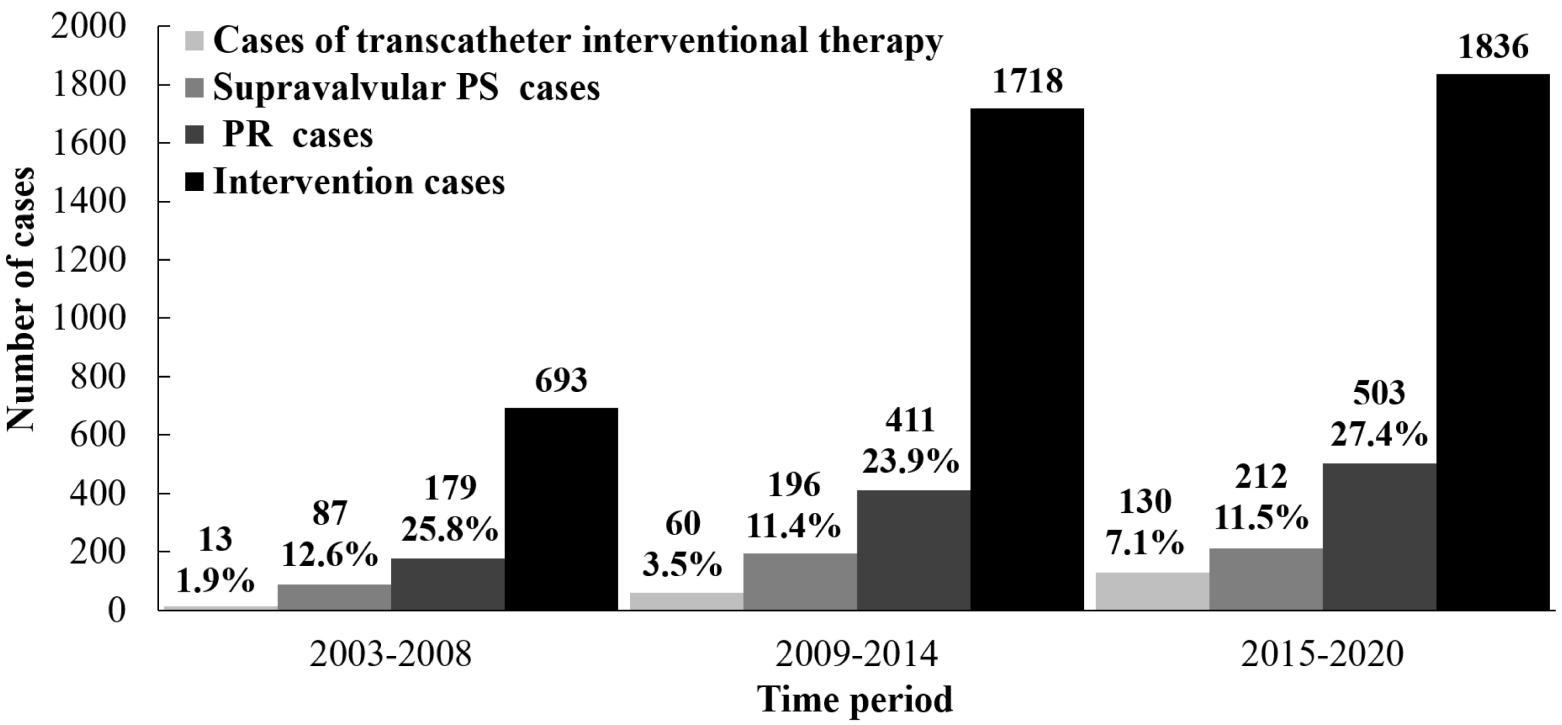
**The change trend in RVOTO patients receiving intervention over time**. In RVOTO cases receiving intervention, over time, the proportion and number of patients receiving transcatheter interventional therapy increased; the number of cases with PR and residual supravalvular/peripheral PS after intervention increased. PS, pulmonary stenosis; PR, pulmonary regurgitation; RVOTO, right ventricular outflow tract obstruction.

**Fig. 6.**
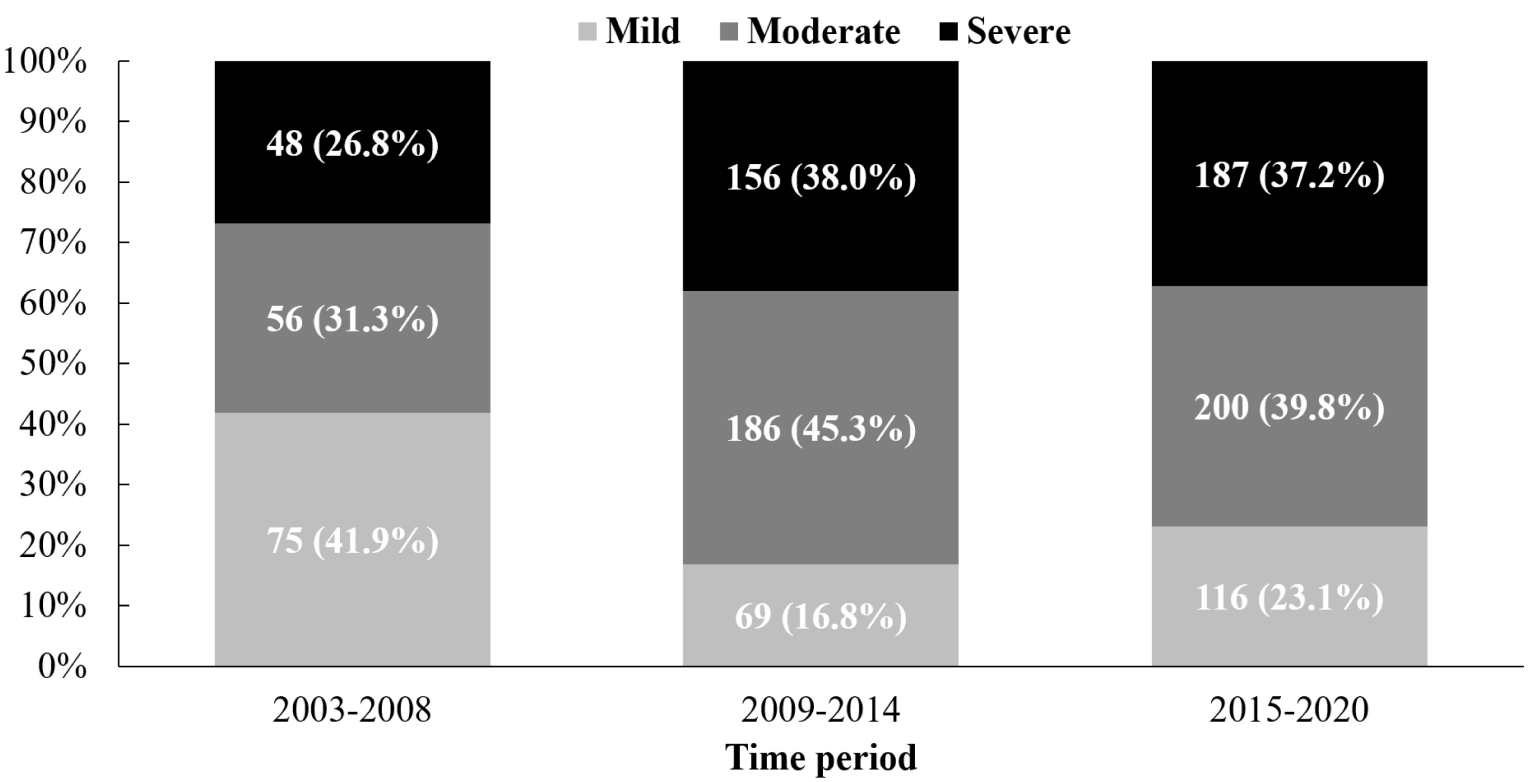
**The increase in the number of pulmonary regurgitations after intervention over time**. The values before the parentheses represent the absolute number of observations of this type, and the values in parentheses represent the proportion of cases of this type in the total cases with pulmonary regurgitation after intervention in each period.

In each time period, the proportion and number of RVOTO cases receiving intervention (surgical or transcatheter) increased (33.1%, 45.1% and 42.4%, respectively, χ^2^ = 31.974, *p* < 0.001; 693, 1718 and 1836 cases, respectively) (Fig. 4). In RVOTO patients receiving intervention, over time, the proportion and number of cases receiving transcatheter interventional therapy (that is, PBPV) increased (1.9%, 3.5% and 7.1%, respectively, χ^2^ = 38.462, *p* < 0.001; 13, 60 and 130 cases, respectively); the number of all cases with PR and residual supravalvular PS after intervention increased (PR: 179, 411 and 503 cases, respectively; residual supravalvular PS: 87, 196 and 212 cases, respectively) (Fig. 5). In patients with PR after intervention, the number of cases with severe PR also increased (48, 156 and 187 cases, respectively) (Fig. 6). It is noteworthy that the number and proportion of patients undergoing re-operation after RVOTO intervention were very low (residual supravalvular PS: 0/87, 0/196 and 3/212; severe PR: 0/48, 0/156 and 1/187, respectively, in three time periods).

## 4. Discussion 

We found that the clinical characteristics of RVOTO have changed significantly over the past two decades: (1) the proportion of RVOTO cases and that of congenital etiology decreased, while the proportion of cases with simple congenital RVOTO increased, and valvular PS, as the most common simple congenital RVOTO, replaced Fallot group as the most common type of RVOTO; (2) the number of RVOTO cases receiving intervention (especially transcatheter therapy) increased, and VSD patch or occluder protruding into RVOT consistently ranked first among iatrogenic causes in RVOTO; (3) the number of cases with severe PR and residual supravalvular PS increased after intervention, but the re-operation proportion was very low.

Currently, the evolution of clinical features of CHD has been studied [29], but data on the prevalence and clinical characteristics of RVOTO remain limited in China [22]. In this large-scale echocardiographic database-based study, we found that the proportion of RVOTO cases decreased over time, but the case number still increased, which may be due to improved accessibility of echocardiography in the general population. The higher proportion of male RVOTO cases, although the gender gap declined over time, may not reflect the actual prevalence of males and females in RVOTO population. Noteworthy is that the current study found an increase in the number of RVOTO cases and a decrease in the proportion of congenital etiology, which may result from the development of prenatal diagnostic techniques and artificial termination of many severe RVOTO fetuses. In addition, acquired RVOTO (mainly due to iatrogenic etiology and other causes including hypertrophic cardiomyopathy) also deserves more attention.

Consistent with previous studies of the prevalence of CHD [20, 21, 22], this study showed that the proportion of cases with simple congenital RVOTO increased over the past two decades and that valvular PS was the most common simple congenital RVOTO and Fallot group still the most common complex congenital RVOTO, with the former replacing the latter as the most common RVOTO. We also found that more than half of the patients in the three time periods were aged ≤3 years old, most of who were ≤1 year old. As most patients receiving treatments were less than 1 year old, it surfaces as a burden and challenge to the long-term care of family and society. Equally noteworthy is that the proportion and numbers of RVOTO patients older than 40 years old increased over time. A previous study has also shown an increasing trend in adult CHD with improved medical conditions [30].

In recent years, advances have been achieved in RVOTO reconstruction surgery. Our data showed that over time, more patients with RVOTO received surgical RVOTO reconstruction or transcatheter therapy (PBPV). Of note, after RVOTO intervention, the number of cases with severe PR and residual supravalvular PS increased over time and the re-operation proportion was very low. The explanation for such a low re-operation rate may be multi-faceted and complex: Some patients have not yet displayed obvious clinical symptoms, presenting no indication of re-operation for the time being; for those with symptoms, they often complain of the risk of re-thoracotomy; the symptoms of such patients tend to progress slowly, with fewer acute symptoms; and despite the recent availability, the current popularity of less invasive technologies (transcatheter valve replacement and stent implantation devices) remains low [31]. Importantly, these severe PR and residual supravalvular PS cases may benefit from PPVI or pulmonary stent implantation. With the development of these interventional technologies in China [14, 15, 19], the popularity of these less invasive technologies will be improved.

Some limitations remain in our study. First, since this is a single-center retrospective descriptive study based on an echocardiographic database rather than the general population, the results may not be suitable for extrapolation to other regions due to different medical conditions. Second, we did not grade the severity of all types of RVOTO, so further research may try to stage the severity degree. Third, due to the large time span, there are some differences in diagnostic standards and equipment, which may exert a mild overall impact. Finally, this study did not evaluate the specific data such as right ventricular volume and cardiac function of patients with RVOTO after surgery. If it is necessary to determine whether patients with severe PR and residual supravalvular PS urgently need surgical treatment, further evaluation is required.

## 5. Conclusion

This echocardiography database-based single-center study demonstrates that clinical characteristics of RVOTO have significantly evolved over the past two decades. Although the overall proportion of RVOTO cases has decreased, the absolute number has increased. RVOTO remains predominantly a pediatric condition, however, we have observed a significant and gradual increase in the proportion of middle-aged and elderly patients, highlighting the growing impact of RVOTO across the lifespan.

Valvular PS has now emerged as the dominant subtype. The study underscores that congenital causes have consistently accounted for the majority of RVOTO cases, with a notable trend towards an increased proportion of simple congenital RVOTO. A critical and concerning finding is the significant rise in iatrogenic causes, which demands heightened clinical vigilance and investigation into contributing factors.

Furthermore, the widespread adoption of RVOTO interventions has unmasked new clinical challenges: a demonstrable increase in cases burdened by severe PR and residual supravalvular PS. This evolving complication profile urgently calls for focused research and the development of innovative solutions, whether these be refined interventional techniques, improved prosthetic valves, or novel therapeutic approaches.

## Availability of Data and Materials

All data included in this study are available upon request by contact with the corresponding author.
